# Interactions of Chromatin with the Nuclear Lamina and Nuclear Pore Complexes

**DOI:** 10.3390/ijms242115771

**Published:** 2023-10-30

**Authors:** Yuri Y. Shevelyov

**Affiliations:** Laboratory of Analysis of Gene Regulation, National Research Centre “Kurchatov Institute”, Kurchatov Sq. 2, 123182 Moscow, Russia; shevelev@img.ras.ru

**Keywords:** nuclear lamina, nuclear periphery, nuclear envelope, lamin, LAD, TAD, heterochromatin, nuclear pore complex, nucleoporin, Elys

## Abstract

Heterochromatin and euchromatin form different spatial compartments in the interphase nucleus, with heterochromatin being localized mainly at the nuclear periphery. The mechanisms responsible for peripheral localization of heterochromatin are still not fully understood. The nuclear lamina and nuclear pore complexes were obvious candidates for the role of heterochromatin binders. This review is focused on recent studies showing that heterochromatin interactions with the nuclear lamina and nuclear pore complexes maintain its peripheral localization. Differences in chromatin interactions with the nuclear envelope in cell populations and in individual cells are also discussed.

## 1. Introduction

In eukaryotic cells, the nucleus is separated from the cytoplasm by the nuclear envelope (NE), which is composed of inner and outer nuclear membranes with a perinuclear space between them. The nuclear lamina (NL), a meshwork of lamins and lamin-associated proteins, lines the inner nuclear membrane. It is anchored in the inner nuclear membrane by nuclear envelope transmembrane proteins [1]. Nuclear pore complexes (NPCs) that penetrate both nuclear membranes are made up of nucleoporins (Nups). NPCs ensure macromolecular transport between the nucleus and the cytoplasm [2].

Chromatin is divided into condensed heterochromatin and less condensed euchromatin. Constitutive heterochromatin covers pericentromeric and telomeric regions of chromosomes, while facultative heterochromatin is represented by islands of inactive chromatin within chromosome arms. Heterochromatin is known to be depleted in histone acetylation, as well as in other active histone marks, and enriched with H3K9me2/3 or H3K27me3 histone modifications [3,4]. Early electron microscopy observations have shown that chromatin is not randomly distributed throughout the nucleus. In most cell types, heterochromatin is located at the nuclear periphery in close association with the NE as well as around the nucleoli, whereas euchromatin occupies a more interior position (see, for example [5,6]). These observations gave rise to the idea that heterochromatin may be linked to the NE, which serves as a scaffold for its tethering [7].

With the advent of new technologies, such as DamID [8], genomic regions interacting with the NL, the lamina-associated chromatin domains (LADs), were identified in various organisms [9,10,11,12]. LADs, which represent peripheral heterochromatin, have a median length of ~0.5 Mb in mammals and ~90 kb in *Drosophila*. Surprisingly, in both organisms, LADs constitute ~40% of the genomes [9,12]. In line with the localization at the nuclear periphery, LADs consist of transcriptionally inactive chromatin corresponding to silent or weakly-expressed tissue-specific genes [9,10,11,12]. It should be mentioned that not all heterochromatin interacts with the NL. For example, in *Drosophila* Kc167 cells, Polycomb (Pc) domains enriched with H3K27me3 only partially overlap with LADs [3].

Apart from the NL, DamID studies in *Drosophila* indicate that NPCs also interact with numerous genomic sites scattered throughout the genome [13,14]. The question then arises about which type of interaction is responsible for the positioning of heterochromatin at the NE.

## 2. Mechanisms of Chromatin Interactions with the NL

Disruption of the NL components results in heterochromatin displacement from the nuclear periphery to the nuclear interior [15,16,17,18,19,20,21], thus indicating that, in normal cells, heterochromatin is attached to the NL. Various proteins of the NE are involved in this attachment [22]. This list includes but is not limited to lamins [19,20,21,22,23,24,25], the lamin-B-receptor (LBR) in mammals (but not in *Drosophila*) [21,25,26], LAP2, emerin, and the MAN1 (LEM) domain proteins in mammals and *Drosophila* [27,28,29,30,31,32], PRR14 in mammals [17], CEC-4 in *C. elegans* [33], and NE transmembrane proteins in mammals [34,35]. These proteins interact with chromatin either directly (such as lamins, LBR, and CEC-4) or through chromatin binding proteins, such as BAF [36,37,38], cKrox with HDAC3 [39], or HP1 [17,26,40,41]. The NE protein PRR14 directly binds H3K9me2/3 chromatin domains, whereas CEC-4 and LBR bind them through HP1α [17,26,33,42]. As a result of this binding, H3K9me2-modified heterochromatin forms a peripheral layer beneath the NL in various organisms [43].

Numerous FISH data indicate that inactive loci are usually removed from the NE upon activation [15,44,45,46,47,48,49,50,51]. These microscopy observations were confirmed by DamID analysis, which showed that most (but not all) activated loci lost contact with the NL [11,52,53]. Loss of contact was most pronounced at the promoters of these loci.

Then what is the mechanism of the detachment of activated loci from the NE? The binding of transcriptional activators, possessing the acidic domain, to the silent locus resulted in chromatin decondensation and locus repositioning to the nuclear interior, even in the absence of locus transcription [54]. The relocalization of loci from the NE to the nuclear interior is likely mediated by a non-diffusion process since nuclear actin and nuclear myosin were shown to be involved [55,56,57,58,59]. It is supposed that the binding of transcriptional activators to the promoter regions recruits the myosin motor to the activated loci, and then it moves loci along the nuclear network consisting of short dynamic actin polymers [60]. However, the mechanism of directionality during this movement is still unclear.

Chromatin decondensation may be induced also by histone acetylation [61,62,63,64]. Thus, the question arises whether histone acetylation by itself leads to the detachment of loci from the NL? A recent study in *C. elegans* demonstrates that artificially increased H3K27 acetylation within silent chromatin domains drives the relocalization of these domains to the nuclear interior [65].

## 3. Mechanisms of Chromatin Interactions with NPCs

There is a growing body of evidence showing that chromatin interacts with various Nups. Studies on yeast performed on several model loci have indicated that, upon induction, these loci moved to the NPCs [66,67,68,69,70]. However, in metazoans, mobile Nups are not only the constituents of NPCs but are also present in the nucleoplasm [71,72]. Moreover, numerous chromatin interactions with Nups have been shown to take place in the nucleoplasm [13,73,74]. For this reason, in the majority of studies in mammals and *Drosophila*, chromatin interactions with Nups identified genome-wide [73,74,75,76,77,78] were not subdivided into those occurring at the NPCs or those occurring in the nucleoplasm. Pioneering work, where such interactions were classified as nucleoplasmic or NPC-linked, was carried out using the DamID technique in *Drosophila* Kc167 cells, which expressed either Nup98 lacking the N-terminus responsible for its association with NPCs or chimeric protein consisting of the N-terminal part of Nup98 and of the integral membrane protein NDC1 [13].

Elys is the only known Nup that possesses chromatin-binding activity [79,80,81,82,83]. Thus, it seems likely that Elys may directly bind chromatin both at NPCs and in the nucleoplasm [84]. Using the ChIP-seq approach with antibodies against stable or dynamic Nups, binding sites of several Nups, including Elys, were identified in *Drosophila* larvae brain and S2 cells [85,86]. Recently, Elys binding sites were also identified in late *Drosophila* embryos using the DamID technique [14]. Almost all Elys sites from embryos were classified as nucleoplasmic or NPC-linked [14] after comparing them with the data from Kalverda et al. [13]. It turned out that NPC-linked sites are mainly represented by short stretches of inactive chromatin embedded in LADs or Pc-domains, whereas nucleoplasmic sites contain active chromatin enriched in the acetylated histone modifications [14,85,86]. Interestingly, nucleoplasmic Elys fraction binds highly acetylated chromatin of enhancers and promoters, and this binding leads to decondensation and “opening” of their chromatin [14,86,87]. Therefore, unlike in yeast, where NPC-linked loci are associated with active gene expression, NPC-linked sites in *Drosophila* mostly correspond to the inactive peripheral heterochromatin [14]. However, using a modified DamID approach, it was recently shown that, in mammals, super-enhancers are associated with the NPCs [88].

**Figure 1:**
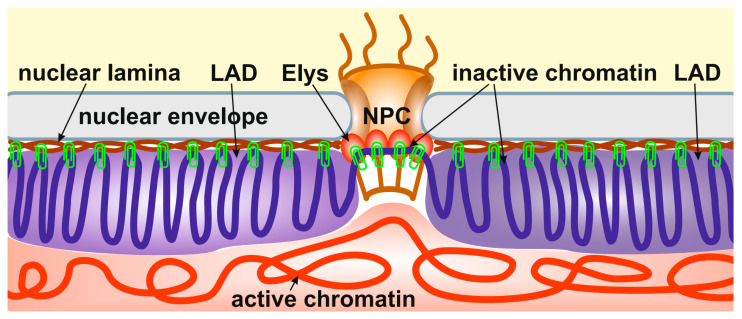
Peripheral heterochromatin is attached through multiple sites to both the NL and NPCs. The schematic shows the anchoring of inactive peripheral heterochromatin (**violet**) at the NL (**brown**) and NPCs (through Elys, **red ovals**). Active chromatin is indicated by **red**. The binding sites of heterochromatin to the NL and NPCs are shown by **green clips**.

Importantly, the depletion of Elys in S2 cells, which does not cause the disappearance of nuclear pores, results in the relocalization of peripheral heterochromatin from the NE to the nuclear interior [14]. A similar effect was observed upon the depletion of lamin Dm0 in S2 cells [21]. Therefore, it can be concluded that heterochromatin is attached through multiple sites to both the NL and NPCs (Figure 1). In support of this model, Elys was shown to interact more strongly with the single X chromosome of *C. elegans* males than with two X chromosomes of hermaphrodites, which correlates with a more intimate association of the male X chromosome with the NE [89].

It should be noted that various Nups can interact with chromatin indirectly through association with chromatin binding complexes. For example, Nup93 was coimmunoprecipitated from protein extracts of *Drosophila* S2 cells together with Pc and the enhancer of zeste (E(z)) [86]. These associations may explain the strong overlap between Nup93-binding sites and Pc-response elements (PREs) in the genome of S2 cells [86]. Nup153 was shown to physically associate with cohesin and CCCTC-binding factor (CTCF), as well as with acetyltransferase CBP/p300 in mammals [90,91]. Nup98 may be recruited to chromatin through its association with the MBD-R2 DNA-binding protein of the nonspecific lethal (NSL) complex or via the trithorax (Trx) complex [92,93]. Apart from these possibilities, Nups can be recruited to chromatin by other Nups [87,94] which additionally complicates the final picture.

## 4. An Influence of Chromatin Detachment from the NE on Genome Architecture

It was found that cell senescence [95,96,97,98], aging [99,100], some human diseases, including Hutchinson–Gilford progeria syndrome [101], as well as rare cases of terminal cell differentiation [25] correlate with the lack of lamins or other components of the NL. The loss of these components is frequently accompanied by heterochromatin relocalization from the nuclear periphery to the nuclear interior. In the case of oncogene-induced cell senescence, peripheral heterochromatin is aggregated into senescence-associated heterochromatin foci (SAHF) in the nuclear interior [18,102]. Cells undergoing replicative senescence rarely form SAHF. Nevertheless, they have lost the peripheral heterochromatin layer [103]. Upon aging, peripheral heterochromatin is also lost in the *Drosophila* fat body, which is the immune organ of this organism [100]. Likewise, fibroblasts from patients with Hutchinson–Gilford progeria syndrome do not have SAHF, but still have a thinner layer of peripheral heterochromatin [104,105]. In the rod photoreceptor cells of animals with nocturnal vision, the 3D genome organization becomes “inverted” [16]. It is characterized by the aggregation of all heterochromatin into a spherical structure in the center of the nucleus, whereas active chromatin appears at the nuclear periphery [16].

Therefore, the emerging picture is that upon the loss of attachment to the NE, heterochromatin is removed from the NE and tends to self-aggregate in the center of the nucleus. Depending on the degree of heterochromatin enrichment with HP1/H3K9me2/3 complex, it may form a common chromocenter, as in rod photoreceptor cells of animals with nocturnal vision lacking both lamin A/C and LBR [16,25], or be only slightly displaced from the NE, as in S2 cells upon lamin or Elys depletion [14,21]. Computer simulations of the nucleus that lost heterochromatin attachment to the NE confirm this model [106]. Another parameter is likely to be the period of time. The complete formation of heterochromatin aggregates in the center of the nucleus requires several weeks without cell division [16].

## 5. Chromatin Interactions with the NL and NPCs in Individual Cells

Interactions of chromatin with the NL and with NPCs were identified in cell populations and, thus, represent the sum of interactions detected in all individual cells. Are these interactions the same in each cell? The current view is that this is not the case. For example, LADs identified in cell populations occupy roughly half of the genome [9,12], calling into question that such a large proportion of chromatin may be simultaneously located at the NE in a cell. Recent mapping of LADs performed in individual cells confirmed that only a small proportion of all LADs (~15–30%) identified in cell population interact with the NL in each cell [107,108].

LADs were classified as constitutive (cLADs) or facultative (fLADs) by their presence in the majority or minority of cell lines analyzed [109]. Consistent with this classification, upon single-cell analysis, cLADs that were shared across cell populations were found to interact with the NL in the majority of individual cells, whereas fLADs interacted with the NL only in a minor fraction of cells [108]. LADs that were localized by microscopy at the NE before mitosis may appear inside the nucleus after mitosis [107]. However, since cLADs exist at a single-cell level, the redistribution of LADs during mitosis is not entirely stochastic. It was noticed that cLADs are enriched with H3K9me2/3 heterochromatin marks in comparison with fLADs [108]. These histone modifications may mediate a stronger and more robust association of cLADs with the NL.

Nevertheless, many LADs appear to be located in the nuclear interior [107,108]. This finding is in agreement with numerous FISH observations showing that many signals corresponding to LADs were detected at some distance from the NE [9,11,15,110]. Moreover, upon 3D-reconstruction of the whole X chromosome after single-cell Hi-C analysis, inactive chromatin, corresponding to LADs identified in cell populations, was rather evenly distributed over the surface of the X-chromosome territory [111] and, thus, was unable to interact with the NL simultaneously from both sides of the chromosome. It should be mentioned that a fraction of LADs located in the nuclear interior may interact with the nucleoli [112,113,114]. However, a notable fraction of LADs in individual cells does not interact with either the NL or nucleoli.

**Figure 2:**
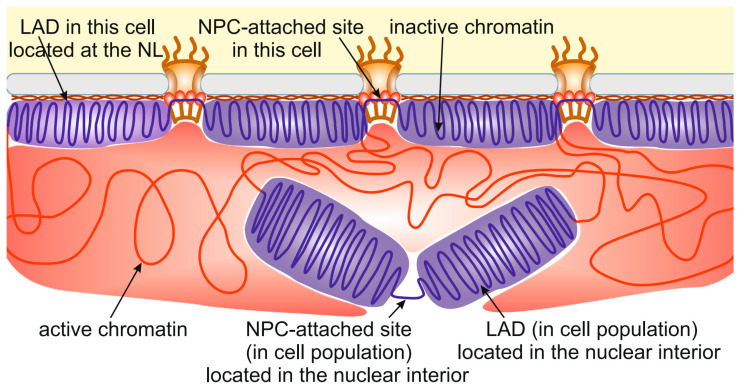
Many LADs identified in cell populations are located in the nuclear interior. The schematic shows the localization of some LADs (**violet**) and NPC-attached sites distantly from the NL (**brown**) and NPCs, respectively. Active chromatin is indicated by **red**.

The same story occurs with chromatin interactions with NPCs. Approximately 4000 NPC-interacting sites have been identified in late *Drosophila* embryos [14]. At the same time, the embryonic S2 cell line has ~1000 nuclear pores per nucleus [115], which is several-fold less. The most plausible explanation is that only a small proportion of all NPC-interacting sites are attached to NPCs in individual cells. Therefore, many LADs and NPC-interacting sites identified in cell population are, in fact, located in the nuclear interior (Figure 2). This may be caused by the competition between different LADs or NPC-interacting sites for binding with the NL or NPCs, respectively. In other words, the limited surface of the NL and the limited number of NPCs do not allow all potential LADs and NPC-binding sites to interact with them. Several indications supporting this idea have been obtained recently. For example, DamID interactions between the NL and chromosomes in the KBM7 cell line, existing in either the diploid or haploid state, were more pronounced in haploid than in diploid cells [108]. Similarly, more interactions with the NL were revealed for the single X chromosome than for each of the paired autosomes in *Drosophila* male germ line cells [53].

Another interesting question is whether LADs interact with the NL along their whole length. The segregation of topologically associating domains (TADs) in *Drosophila* was shown to follow epigenetics, i.e., some long TADs are collinear to LADs, while some short TADs are collinear to active genes or gene clusters [116,117]. It is hard to imagine how TADs consisting of inactive chromatin, which are visualized by super-resolution microscopy as 3D globular structures [118,119], can be attached to the NL simultaneously by all nucleosomes. Rather, the collinearity between inactive TADs and LADs does exist in a cell population, while only a fraction of nucleosomes from each inactive TAD interacts with the NL in a single cell.

## 6. An Influence of Chromatin-NE Interactions on Chromatin Compaction

Early cytological observations indicated that heterochromatin is more compact than euchromatin [120]. Mammalian LADs have been shown to be enriched with the H3K9me2/3 mark through their whole length and with the H3K27me3 mark at LAD boundaries [9,121]. In the differentiated cell types of *Drosophila*, such as neurons, LADs are also enriched with H3K9me2/3 [122]. H3K9me2/3 and H3K27me3 histone modifications are the targets for binding of HP1 and Pc repressors, respectively [40,41,123,124]. It has been shown that both HP1 and Pc were able to condense chromatin [125,126,127,128]. Thus, their binding to LADs is one of the reasons for the compact state of peripheral heterochromatin. However, LADs in embryonic *Drosophila* Kc167 cells are not enriched with H3K9me2/3, and only half of them overlap with Pc-domains [3,12]. Yet, they strongly overlap with histone H1 [3], which is able to compact chromatin [129].

Using the technique of chromosome conformation capture with high-throughput sequencing (Hi-C, [130]), two recent studies have analyzed the impact of detachment of LADs from the NL on their chromatin compaction [20,21]. Loss of all lamins in mouse embryonic stem cells resulted in the decreased packaging density of a fraction of NL-attached TADs [20]. Similarly, the average frequency of Hi-C contacts in TADs, calculated before and after the loss of all lamins in *Drosophila* S2 cells, has shown that TADs corresponding to LADs became less compact upon NL disruption. Thus, in cells possessing intact NL, interactions of LADs with it result in the elevated chromatin compaction [20,21]. Moreover, computer modeling has shown that NE attachment, by itself, leaded to the increased chromatin packaging density [21]. Therefore, besides HP1- or Pc-mediated compaction, LADs become mechanically more compact due to their attachment to the NL.

Mechanosensing and mechanotransduction in cells are mediated by the linker of the nucleoskeleton and cytoskeleton (LINC) complex [131]. It is composed of the KASH domain proteins (nesprins in mammals) integrated into the outer nuclear membrane and protruding into the cytoplasm, which are bound with the SUN domain proteins integrated into the inner nuclear membrane [132,133]. KASH proteins, which interact with various cytoskeletal components, receive cytoplasmic signals and transmit them to the nucleus through the NL-associated SUN proteins [134,135]. It is currently accepted that mechanical rigidity of nuclei is determined by both the NL and chromatin (for example, [136]). In support of the role of chromatin in this process, chromatin decondensation has been shown to result in a more profound deformation of nuclei in response to extracellular forces, while chromatin condensation, on the contrary, enhanced the stiffness of nuclei [136,137]. Chromatin decondensation may be caused by the loss of chromatin attachment to the NE. However, it is unclear whether the attachment of heterochromatin to the NE *per se* makes the nuclei more rigid. A recent study on yeast has shown that heterochromatin attachment to the NE does matter [138]. Upon depletion of LEM-domain proteins that tether heterochromatin to the NE, the rigidity of nuclei was decreased. Therefore, the intactness of the NL, the degree of chromatin compactness, and interactions between the NL and chromatin are the major determinants of the stiffness of nuclei that resist extracellular forces.

What is the influence of chromatin attachment to NPCs on its compaction? To address this question, Hi-C analysis was performed in S2 cells depleted for Elys, which is responsible for peripheral heterochromatin tethering to the NPCs [14]. It was found that, upon Elys loss, TADs consisting of inactive chromatin became less compact, while active TADs became more compact [14]. It seems likely that, upon Elys loss, the less compact state of TADs, which correspond to LADs, is mediated by the lack of interactions between LADs and the NL [14]. Interestingly, chromatin tethering to NPCs through Elys facilitates Hi-C contacts between chromatin regions locally adjacent to the site of attachment [14].

Elys is known to be present also in the nucleoplasm, where it binds active, acetylated chromatin [14,85,86]. Artificial recruitment of Elys to several sites on *Drosophila* polytene chromosomes results in their decompactization [87], which is likely caused by the PBAP chromatin-remodeling complex associated with Elys [139]. Therefore, a more compact state of TADs consisting of active chromatin upon Elys depletion can be explained by the lack of PBAP, normally recruited by Elys to the active promoters and enhancers [14].

## 7. An Influence of Chromatin-NE Interactions on Gene Expression

Currently, it becomes clear that interactions of loci with the NL only weakly affect gene expression. For example, artificial tethering to the NL resulted in a two- to three-fold down-regulation of the low-expressed reporter genes as well as of endogenous genes located near the sites of tethering [140,141] but had a subtle effect on the strongly-expressed reporter genes [140,142]. Moreover, the disruption of the NL resulted in only two- to three-fold transcriptional up-regulation of silent genes but did not notably change active gene expression [15,21]. By using single-cell DamID coupled with a single-cell RNA-seq in mouse embryonic stem cells, it was recently revealed that when a locus interacts with the NL in a cell, its median expression level is about two-fold lower than when it is detached from the NL in another cell of the same population [143]. However, various TRIP experiments have shown that reporter gene expression appeared to be significantly lower when the reporter was integrated into LADs, as compared to inter-LADs [144,145]. The explanation for this discrepancy may be the following. LADs contain chromatin, which exerts a strong repressive effect on the integrated reporter gene [145]. However, this effect is mainly mediated by the presence of silencers within LADs but not by the attachment of LADs to the NL.

It should be mentioned that the influence of the NL on the repression of attached genes may be stronger in the differentiated cells [35,122,146]. In mouse embryonic stem cells, interactions of LADs with the NL are less pronounced than in more differentiated cells, and this effect correlates with the more abundant presence of H3K9me2/3 modification in the latter case [11,121]. In *Drosophila* neurons, LADs are enriched with HP1 binding, which is absent in embryonic Kc167 cells [122]. The binding of HP1 to H3K9me2/3, which makes chromatin more condensed [127,128] and, thus, enhances gene repression in the neuronal LADs [122], may be stabilized by the association of H3K9me2/3-modified LADs with the NL. For this reason, NL disruption in the neurons potentially may have a more severe derepression effect on the NL-attached genes. Similarly, muscle-specific expression of some NETs in mouse cells results in the stronger binding of a set of myogenic genes with the NL accompanied by the enhanced repression of these genes [35].

A recent study indicates that depletion of Elys in *Drosophila* S2 cells, leading to the partial detachment of peripheral heterochromatin from the NE, also barely affects gene expression [14]. The two-fold transcriptional up-regulation, detected for silent or weakly expressed genes, is likely caused by partial loss of interactions between LADs and the NL [14]. Therefore, heterochromatin tethering to the NL and NPCs causes suppression of the background transcription of silent genes.

In addition, tissue-specific up-regulation of Nups during differentiation may affect the expression of a subset of genes more drastically. For example, the inclusion of tissue-specific transmembrane Nup210 in NPCs during myoblast differentiation is required for efficient expression of muscle-specific genes. Nup210 recruits the Mef2C transcriptional complex to the NPC-attached genes, thus enhancing their expression [147]. Another example is Seh1, a scaffold Nup, which recruits the Olig2-dependent transcriptional complex to NPCs to promote oligodendrocyte differentiation in mammals [148].

Moreover, the attachment of some inducible genes to the NPCs may mediate transcriptional memory, i.e., more rapid activation of their transcription in response to repeated treatment by external stimuli [85,149,150,151,152]. The mechanistic model explaining this phenomenon is based on the idea that communication between enhancer and promoter may be stabilized by anchoring both elements at the same NPC [85,150]. Nevertheless, transcriptional memory can occur at some loci located in the nuclear interior [151] or relocated to the nuclear interior upon gene induction [91]. Therefore, the detailed mechanism of this phenomenon awaits further clarification.

## 8. Conclusions

We now realize that analysis of the genome architecture in cell populations and at a single-cell level draws different pictures of chromatin–NE interactions. Although cell population studies indicate that roughly half of the genome may interact with the NL, no more than 15–30% of these LADs are attached to the NL in each individual cell [107,108]. A similar proportion of genomic sites that are potentially able to interact with NPCs are tethered to NPCs in each cell. Therefore, many LADs with NPC-interacting sites are located in the nuclear interior. This understanding leads to the important conclusion that, at least in embryonic cells, the attachment of genes to the NL or NPCs can not strongly affect their expression because the list of attached genes varies from cell to cell.

Another important conclusion is that heterochromatin is maintained at the nuclear periphery through the multiple interactions with the NL, interspersed with its interactions with the NPCs. Without each type of interaction, heterochromatin relocates from the NE to the interior of the nucleus.

## Data Availability

Not applicable.
